# Kinetic Effects of 6 Weeks’ Pilates or Balance Training in College Soccer Players with Chronic Ankle Instability

**DOI:** 10.3390/ijerph191912903

**Published:** 2022-10-08

**Authors:** Quan Jiang, Yonghwan Kim, Moonyoung Choi

**Affiliations:** 1 Department of Public Sports, Luoyang Normal University, Luoyang 471934, China; 2 Department of Physical Education, Gangneung-Wonju National University, Gangneung 25457, Korea; 3 Department of Sports Science Convergence, Dongguk University, Seoul 04620, Korea

**Keywords:** rehabilitation, Pilates, balance training, chronic ankle instability, lateral ankle sprain, strength, dynamic balance, kinetic

## Abstract

Lateral ankle sprain (LAS) is a common sports injury that frequently occurs in active individuals. LAS is characterized by a high recurrence rate, with a large proportion of patients progressing to chronic ankle instability (CAI). Pilates exercises have provided positive results in health care and in rehabilitation. This study compared Pilates training (PT) with traditional balance training (BT) in patients with CAI. Fifty-one college football players with CAI, divided into PT (*n* = 26) and BT (*n* = 25) groups, were included in the study. The groups performed PT or BT training as assigned, three times per week for 6 weeks. Isokinetic ankle strength, one-leg hop tests, Y-balance test (YBT), and foot and ankle outcome score (FAOS) were evaluated before and after training. There were considerable improvements in both the PT and BT groups after training. Group and time comparisons revealed that the PT group achieved better triple hop test results than the BT group, whereas the BT group exhibited a greater improvement in YBT posteromedial and posterolateral reach distances. In athletes with CAI, both PT and BT effectively improved symptoms and function. These findings suggest that ankle strength, balance, and core stability should be comprehensively evaluated and targeted in CAI rehabilitation programs.

## 1. Introduction

Lateral ankle sprain (LAS) is the most common sports injury in athletes, and 60% of high school and college athletes have experienced at least one LAS [1,2]. Although LAS is most commonly reported in competitive athletes, a very high prevalence has also been observed in the general population [3]. LAS impacts various aspects of physical activity and can significantly affect health-related quality of life [4]. The International Ankle Consortium defines LAS as an acute traumatic injury to the lateral ligament complex of the ankle due to excessive inversion of the rear foot or combined plantar flexion and inversion of the foot [5]. LAS is characterized by a high recurrence rate, and a considerable number of patients progress to chronic ankle instability (CAI) [6].

In a previous study, 30–40% of individuals who experienced LAS complained of subjective ankle instability that persisted even after time had elapsed, and it has been reported that they progressed to CAI within 1 year of the initial sprain [1]. Patients with CAI typically present with recurrent ankle sprains as a result of mechanical instability (pathological relaxation after ankle ligament injury) and functional instability (proprioceptive deficit and neuromuscular dysregulation) [5,7]. CAI is characterized by repeated perception of the ankle giving way and presents with low self-reported function and symptoms such as general weakness of the ankle, pain, limited range of motion (ROM), and decreased function. These problems contribute to impairments associated with reduced health-related quality of life and deficits in sensory–motor control [8].

To date, numerous interventions have been investigated to ameliorate mechanical and functional defects associated with CAI. Among them, various studies have proven that rehabilitation is an effective nonsurgical intervention to improve defects related to functional instability. Most studies have focused on improving ROM, eversion strength, dynamic balance, proprioception, and neuromuscular control that was directly applied to the ankle [9,10,11,12,13]. Improvement of these biomechanical and functional factors is ultimately associated with the restoration of ankle strength, peroneal reaction time, and postural stability to pre-LAS levels in patients with CAI [13].

In particular, postural stability is greatly affected by ankle biomechanics and function, as well as sensory–motor control and core stability. As the core muscles are at the center of the body, they maximize the connections in the upper and lower extremity motor chains that are involved in strength, balance, and movement control. This creates a stable basis for adequate muscle recruitment and timing in sensorimotor control [14,15]. Core stability is related to the body’s ability to control the trunk in response to internal and external disturbances. This includes forces generated by distal body parts and the ability to respond to unexpected perturbations [16]. Therefore, it is important to improve core stability to treat persistent ankle pain and instability associated with recurrent sprains after LAS, given the various movements of the body that are required for sports and physical activity.

However, there are not many studies that have applied core training to CAI patients. One study reported that 8 weeks of core training increased the postural stability of CAI patients [17], and a review paper also suggested that core muscles play a positive role in postural stability of CAI patients [18]. Conversely, other studies have published results that show a low correlation between core strength and CAI [19]. These results suggest that further research is needed.

Therefore, we conducted this study because core stability and CAI-related studies are lacking, and the results were different for each study. The research hypothesis was established that core stability training would improve the biomechanical and functional deficits associated with CAI. In this study, Pilates training was applied for core stabilization. Previous studies have reported that Pilates training is effective in stabilizing the core and improving posture control [20,21,22]. This study compared the changes in isokinetic ankle strength, functional hop tests, and dynamic balance, and subjective ankle scores were compared between college soccer players with CAI who received Pilates training (PT) and those who received balance training (BT).

## 2. Materials and Methods

### 2.1. Participants

The required sample size was calculated using G*power software (G*power 3.1, University of Düsseldorf, Düsseldorf, Germany). The conditions were set as follows: F-test and repeated-measures, within-between interaction, effect size f = 0.25, correlation among repeated measures = 0.5, α error = 0.05, power (1-β err prob) = 0.95, number of measurements = 2, number of groups = 2. According to this result, the final number of recruits should have been 54 (PT, *n* = 27; BT, *n* = 27); however, one patient in the PT group and two patients in the BT group dropped out. Therefore, the final analysis included 51 patients (PT, *n* = 26; BT, *n* = 25).

Participants were recruited via the Internet, rehabilitation centers, hospitals, and bulletin boards of universities with soccer clubs. Only university players who expressed interest in voluntary participation after seeing the announcement were included in the experiment. The inclusion criteria were as follows: (1) male college soccer players aged 19–24 years, and (2) experienced LAS in the ankle within the previous one-month period. Athletes who visited the center consulted with a specialist and only those diagnosed with CAI after comprehensive evaluation of X-rays, questionnaires, and physical examinations participated in the study. The allocation of training was based on the players’ preferences. The researchers explained the purpose, advantages, and potential risks of the study. All participants provided written informed consent. The study complied with the Declaration of Helsinki and was approved by the institutional ethics review committee of Gangneung-Wonju University (GWNUIRB 2021-13).

### 2.2. Isokinetic Ankle Strength

Isokinetic ankle strength was measured using an isokinetic dynamometer (Humac Norm, CSMi, Stoughton, MA, USA) to measure ankle inversion (IV), eversion (EV), plantarflexion (PF), and dorsiflexion (DF). An isokinetic dynamometer is a mechanical device that controls the speed of movement so that the muscle can contract at a constant rate while actively resisting the passively applied force in a set joint ROM [23]. To maintain consistent posture during the examination, the participants lay on the examination chair in a supine position, with the hip and knee flexed at 90°; the distal part of the thigh was supported and fixed with a strap. The feet were fixed with cross strips to the ankle test attachments. The axis of rotation of the dynamometer was aligned with the center of the talus when evaluating inversion and eversion and with the lateral malleolus when evaluating plantarflexion and dorsiflexion [24]. In addition, to set the neutral position of the ankle, inversion and eversion aligned the second toe and the center of the tibia, and plantarflexion and dorsiflexion aligned the ankle joint at right angles. The neutral position was defined as the 0° state. Based on the neutral position, the ROM of the ankle for examination was set to 40° for inversion, 30° for eversion, 40° for plantar flexion, and 20° for dorsiflexion. Measurements were performed with concentric contractions of the ankle invertor, evertor, plantar flexor, and dorsiflexor muscles at an angular velocity of 30°/s. To facilitate participants’ understanding of the procedures, an experienced inspector provided demonstrations with adequate explanations of the evaluation methods; several practice exercises were allowed to ensure familiarity before the actual tests were conducted. For each measurement, the participants performed the main test four times, after two practice movements. To enhance accuracy, the uninjured ankle was measured first, followed by the involved ankle. The measured peak torque was recorded in Newton meters (Nm).

### 2.3. Functional Hop Tests

Functional hop tests were performed to evaluate the functional performance of the lower extremities in relation to the ankle. This test is commonly used to evaluate ankle function and includes single, triple, crossover, and 6-m timed hop [25]. All tests were conducted on a course marked with strips on the floor, extending to 6 m. To prevent injury, all participants were provided with sufficient time to warm up before testing. The examiner explained the procedure to the participant, demonstrated the posture, and allowed participants to practice to gain familiarity with the examination procedure. In the single-hop test, participants stood on one leg, jumped as far as possible with maximum effort, and landed on the same foot. In the triple-hop test, the participant performed three consecutive hops in a straight line as far as possible on one leg. In the crossover hop test, the participant alternated to the left and right of a centerline with one leg three times, as far as possible. In the 6-m timed hop test, the participant stood on one leg and passed a 6 m distance by performing a series of consecutive single-leg hops as quickly as possible, without losing balance. The single-, triple-, and crossover hop tests measured the total jumping distance. The 6-m timed hop test measured the time taken to cross the finish line from the starting line using a stopwatch. After performing the test twice on each lower extremity, the better value for each lower extremity was used in the analysis.

### 2.4. Dynamic Balance

Dynamic balance related to postural stability was measured using Y-balance test (YBT) equipment (Y Balance Test™, Chatham, VA, USA). YBT is a dynamic balance test that requires strength, flexibility, stability, and proprioception of the lower extremities including the ankle [26]. After an experienced examiner demonstrated the correct posture and sequence of motions, sufficient practice time was provided to the participants. After the practice session, the participants placed one foot on the stance plate at the center of the equipment in a single-leg stance. The opposite leg was extended as far as possible, and a motion sequence was performed to sequentially push the indicator box in the anterior, posteromedial, and posterolateral directions with the tip of the foot while maintaining balance. During the measurement, if the foot pushing the indicator box touched the floor or the heel of the standing leg fell off the stance plate, remeasurement was performed. The procedure was performed for the three separate directions and measured twice for each lower extremity; the higher value recorded for each lower extremity was used in the analysis. The absolute reach distance measured in each direction was recorded in centimeters.

### 2.5. Subjective Ankle Score

Ankle scores related to the participants’ subjective symptoms and function were measured using the foot and ankle outcome score (FAOS). The FAOS is a clinical-based questionnaire used for the subjective self-assessment of physically active patients with symptoms and functional limitations of the feet and ankles [27]. The FAOS comprises five individual subscales related to foot and ankle pain (nine items), other symptoms (seven items), activities of daily living (17 items), sport and recreation function (five items), and quality of life (four items). It is a questionnaire composed of 42 items in total. Items are scored on a 5-point Likert scale ranging from 0 to 4. The sum of the scores of each individual subscale was normalized to a percentage, and the average score of the five subscales was calculated to obtain the total score. Higher scores indicated fewer symptoms or functional limitations in the feet and ankles, and no limitations in activities or sports. In the data collection procedure, the examiner explained the purpose and contents of the FAOS to participants and provided a pen and questionnaire. The participants completed the questionnaire and were provided with sufficient time and personal space to respond to each subscale.

### 2.6. Training Programs

#### 2.6.1. Pilates Training

The PT program applied the Stott Pilates method, which emphasizes a neutral spine posture and core stability. The PT program was composed by extracting movements suitable for the research purpose from the contents of the various literature [28,29,30]. The movements consist of a neutral spine position and movements that emphasize core stability. It was written mainly for mat Pilates movements that do not use complicated or expensive equipment. The program included core-focused movements during Pilates mat exercises that could be performed without the need for tools and equipment other than floor mats. Participants in the PT group performed the PT program three times per week for 60 min per session (10 min warm-up, 40 min Pilates, and 10 min cool-down).

The program was conducted over a period of 6 weeks and comprised 24 sessions. In Pilates, it is very important to focus on the consistency of the instruction method to provide the same exercise effect to all participants. Therefore, the cuing, sequence, and number of repetitions were determined in advance and applied consistently by the same Pilates instructor. The training intensity, including the difficulty of movements and the number of repetitions, started at the beginner level and gradually increased over time. In addition, 30–60 s of each movement was configured to continuously generate muscle contraction, and the number of repetitions was increased. The training intensity felt by the participants was conducted at the rate of perceived exertion (RPE) 13 to 15 level using the Bog scale 20 scale (Table 1).

#### 2.6.2. Balance Training

The BT program consisted of various balance exercises to improve postural control and proprioception (Table 1). The purpose of the training program was to improve ankle–body coordination and stability through neuromuscular activation. The researchers reconstructed BT to fit the actual situation by referring to the prior literature [12,13,31,32]. The participants performed the BT program for 60 min per session three times a week for 6 weeks (10 min warm-up, 40 min Pilates, and 10 min cool-down), the same as the PT group. The initial training program consisted of exercises performed on the floor, without balance equipment, to provide perturbation. As the training continued and time progressed, balance equipment that made it difficult to control the posture was applied to gradually increase exercise intensity with posture difficulty, repetition, and duration. In this study, a BOSU balance trainer (BOSU, Ashland, OH, USA) was used as balance equipment to provide an unstable surface. First, static and dynamic balancing exercises were performed on the hard flat side of the BOSU, and gradually, to increase the difficulty in controlling the posture, it was performed by standing on the unstable surface of the soft dome side. The intensity of BT training was performed at RPE 13~15 level like PT group.

### 2.7. Data Analysis

Data were analyzed using SPSS Statistics software version 25.0 (IBM Corp., Armonk, NY, USA). Continuous variables are expressed as means and standard deviations using descriptive statistics. Strength, hop tests, YBT, and the FAOS, corresponding to the main variables, were compared before and after training in each group using a paired *t*-test. The ratio of the affected side to the healthy side of each individual was calculated as the limb symmetry index (LSI): limb symmetry index = (injured side/uninjured side) × 100. Repeated two-way analysis of variance was used to test the interaction effect over time within the group. Statistical significance was set at *p* < 0.05.

## 3. Results

### 3.1. General Characteristics of Participants

Participants were classified according to the training group. Table 2 summarizes the general characteristics of the participants. Age, height, weight, and body mass index were not significantly different between the groups. In addition, there was no significant between-group difference regarding the experience of giving way during the last week or month, which indicates LAS or ankle instability.

### 3.2. Isokinetic Ankle Strength

Table 3 shows the changes in ankle strength before and after training, and the differences between the groups. The strength of ankle eversion and dorsiflexion significantly increased after training in both PT and BT groups; however, there was no significant difference in the interaction effect according to time and group. The strength of inversion and plantar flexion did not change significantly with PT or BT. Similarly, for LSI, both groups exhibited significant improvements only in eversion and dorsiflexion strength, with no significant change in inversion and plantar flexion.

### 3.3. Functional Hop Tests

Table 4 presents the changes in the jump test before and after training. In both the PT and BT groups, single, triple, crossover, and 6-m timed hop tests significantly improved distance, time, and LSI after 6 weeks of training. In particular, changes in time and group were significant only for the triple hop task. This means that both groups improved through training, but the PT group improved to a greater extent than the BT group.

### 3.4. Dynamic Balance

The YBT results measured to evaluate dynamic balance are shown in Table 5. In both groups, the reach distance and LSI in the anterior, posteromedial, and posterolateral directions increased considerably after training. The interaction effect was significant only in the posteromedial and posterolateral directions, indicating that BT is more effective in improving dynamic balance than PT.

### 3.5. Subjective Ankle Score

Table 6 shows the subjective ankle scores. In all five subsections included in the FAOS, there was improvement after training compared to before training in both groups. However, there was no interaction effect in the two groups over time.

## 4. Discussion

CAI is caused by LAS, which is one of the most common sports injuries; this study compared various kinetic variables by applying balance training, which has traditionally been performed for CAI, and the recently popularized Pilates training, over a 6-week period.

One of the main results of this study was that ankle eversion and dorsiflexion strength were improved after training in both the PT and BT groups in the isokinetic ankle strength results. These results imply that although only PT or BT training was performed, ankle strength training was effective in improving ankle muscle function in individuals with CAI.

In previous studies, several authors have reported that BT affects balance and also improves ankle strength [13,33]. BT stimulates the muscle fibers and nerves around the ankle during static and dynamic postural control in a closed kinetic chain. In addition, it has been reported that the balance equipment used to provide perturbation affects activation of the ankle muscles, such as the tibialis anterior, peroneus longus, and medial gastrocnemius [34]. Interestingly, improvements in eversion and dorsiflexion strength were also observed in the PT group. However, it was difficult to determine whether this was an effect of training or a result of pain relief and natural recovery over time. Since no control group was established in this study, further studies should be conducted for more clear facts on this issue.

In addition, in both groups, there were significant changes only in eversion and dorsiflexion in this study, and the results were similar to those of a previous study [35]. There was no significant difference between inversion and plantar flexion. This suggests that peroneus muscle weakness occurred in CAI, resulting in decreased strength in eversion and dorsiflexion involving the peroneus muscle, whereas inversion and plantar flexion were less affected [36]. These data also show that the LSI of inversion and plantarflexion before training was approximately 90%, which means that the muscle strength was better than that of the unaffected side.

Hop tests are generally performance-based assessments that measure the functional performance of the lower extremities and are often used to evaluate the level of functional recovery after an ankle injury [37]. In this study, hop tests were conducted based on this background information. In this study, both PT and BT improved significantly after the training. A previous study also showed similar results, in which all four hop tests improved after six weeks of balance training [12]. In a study by Sonepat et al. [38], training using a wobble board for 8 weeks increased the distance of the triple hop test. Similarly, a study by Aslan et al. [39] reported that the distance of the triple hop test was significantly improved after 8 weeks of core training. Although PT and BT does not apply strong resistance, such as strength training, functional improvement would have been achieved by adequately stimulating the muscular nervous system. In this study, an increase in dynamic stability was also observed in both PT and BT, and this improvement in postural stability is believed to have had an effect on the performance improvement of the functional hop tests.

The hop test evaluates a combination of ankle strength, neuromuscular control, confidence in the lower extremities, and ability to withstand loads related to sports-specific activities [25]. Depending on the study, only a single-hop test is performed owing to its simplicity and time efficiency, but since the single-hop test induces and evaluates only ankle stress in the sagittal plane, there is a limitation in evaluating control ability in various directions [37]. Haitz et al. [40] also reported that the sensitivity ratio of the test increased when two or more hop tests were evaluated together compared to when only one hop test was evaluated when assessing function of the lower extremities.

As both PT and BT play a role in improving postural control and stability [13,33,41], it would have been improved in all hop tests in this study as well. Although both groups showed improved results in all hop tests, the interaction effect in the triple hop was perhaps due to the effect of core stability training, which is the main focus of PT. Core stability allows for the optimal generation, transmission, and control of forces and motion of the extremities in integrated kinetic chain activity [15]. The theoretical background of the core stability training effect is as follows: Because core training shortens the amortization phase in the stretch-shortening cycle, it aids the efficient use of the elastic energy stored in the eccentric pre-stretch phase in the subsequent concentric shortening phase [42]. This improved stretch reflex is associated with more effective plyometric performance and is thought to have a greater effect on a similar motion, that is, triple hop.

Dynamic balance refers to the ability to maintain postural stability while moving the body or changing the position of a limb and is an important component in most daily life and sports activities [43]. In this study, dynamic balance was evaluated using the YBT. Both groups that received PT and BT improved significantly, but BT was more effective in posteromedial and posterolateral directions than PT. These results may be attributed to the characteristics of the training program conducted in the BT group in this study. Most BT programs conducted in this study comprised static and dynamic posture control training performed in a single-leg stance. This could have had an effect on the BT group, who showed greater improvement than the PT group.

Proprioception of the ankle maintains postural stability by receiving the joint position information of the extremities via the receptor and rapidly transmitting it to the central nervous system. Proprioception is mainly related to the sense of position of mechanoreceptors, which includes both static and dynamic positions. [44]. Individuals with CAI experience repeated LAS or giving way because these mechanisms are impaired [8,45]. Terada et al. [46] confirmed that patients with CAI showed decreased dynamic balance due to inhibition of tibialis anterior muscle activity in a single-leg stance, and Nanbancha et al. [47] reported a decrease in dynamic ankle stability related to neuromuscular control in patients with CAI.

Subjective self-evaluation using questionnaires is not a kinetic analysis, but the FAOS, which is commonly used in clinical practice, was applied in this study [27]. In this study, the subjective ankle score evaluated by the FAOS was significantly improved after training in both groups that received PT and BT. The improvement in subjective evaluation through training intervention has already reported the same results in previous studies [12,31]. A minimal clinically important difference (MICD) was identified to confirm whether the self-reported results indicated clinically significant changes. The MCID is defined as the smallest change in a measure that indicates a significant improvement in the symptoms of a disease [48,49]. In the present study, after training, both PT and BT groups reported high subjective scores exceeding the MCID. This suggests that PT and BT are effective interventions to improve the subjective pain, symptoms, and function associated with CAI, even in athletes with high activity levels and functional demands.

This study provides useful information that can be used in developing rehabilitation programs. Even if the load is not directly applied to the ankle, core-based Pilates training could improve muscle strength, one-foot hop function, dynamic balance, and improve subjective satisfaction in CAI. The Pilates method defines the core as the “powerhouse” of the body and emphasizes six basic principles: centering, concentration, control, flow, breath, and precision. Pilate progression is achieved by manipulating the effects of the base of support, gravity, length of levers, and center of gravity. Through this process, rapid reaction time, trunk stabilization, and improved postural control can be exhibited [20,50].

Our study had several limitations. We suggested that all participants adhere to the training presented. Moreover, additional individual or team ankle strength, balance, and core training that could affect the study results was indicated to be prohibited. Nevertheless, we did not have a system that fully controlled their exceptional training. In addition, although the female soccer population has been increasing recently, women were not included in this study. Moreover, there is a limit in evaluating the improvement of symptoms and kinetics over 6 weeks because the control group setting without any training intervention was not established. In addition, in this study, PT and BT allocations were not randomly assigned, and participants’ preferences were respected. This was because the opinions of the participants could not be overlooked in ethical research. In future studies, an experiment that compensates for these limitations should be conducted, and it would be very valuable to conduct a more detailed study on the local muscle activation among the core muscles.

## 5. Conclusions

In this study, participants with CAI who performed PT or BT for 6 weeks showed significantly improved results of ankle eversion, dorsiflexion isokinetic strength, functional hop tests, YBT, and the FAOS after training. In the comparison between groups, after training, the PT group showed a greater improvement regarding distance and LSI in the triple hop test, and the BT group showed improved posteromedial and posterolateral reach distances and LSI in the YBT. Both PT and BT are effective training interventions to improve symptoms and function in athletes with CAI. These results suggest that ankle strength, balance, and core stability should be comprehensively evaluated and trained in rehabilitation programs for patients with CAI. In particular, Pilates-based core stability training can be useful as an effective initial training intervention in patients with acute ankle sprains who have limitations in performing advanced ankle strengthening training due to ankle pain and symptoms.

## Figures and Tables

**Table 1:** Pilates training and balance training programs.

	PT	BT
1–2 weeks	Abdominal prep, 10 reps	Balance equipment: None (floor)
	Rolling like a ball, 10 reps	Tandem stance, 30 s × 5 sets
	Hundred, 1 set	Single leg balance, 30 s × 5 sets
	Roll up, 5 reps	Single leg swings (sagittal plane), 20 reps × 3 sets
	Single leg stretch, 5 reps in each leg	Single leg swings (frontal plane), 20 reps × 3 sets
	Single leg circle, 5 reps in each leg	Standing single leg circle, 20 reps × 3 sets
	Shoulder bridge prep, 10 reps	Single leg squat, 20 reps × 3 sets
	Swimming prep, 10 reps	Single leg deadlift, 20 reps × 3 sets
3–4 weeks	Abdominal prep, 10 reps	Balance equipment: None (floor)
	Rolling like a ball, 10 reps	Tandem stance, 60 s × 3 sets
	Hundred, 1 set	Single leg balance, 60 s × 3 sets
	Roll up, 10 reps	Single leg swings (sagittal plane), 30 reps × 3 sets
	Single leg stretch, 10 reps in each leg	Single leg swings (frontal plane), 30 reps × 3 sets
	Single leg circle, 10 reps in each leg	Standing single leg circle, 30 reps × 3 sets
	Shoulder bridge prep, 20 reps	Single leg squat, 30 reps × 3 sets
	Swimming prep, 20 reps	Single leg deadlift, 30 reps × 3 sets
5 weeks	Abdominal prep, 10 reps	Balance equipment: BOSU (plat side)
	Rolling like a ball, 10 reps	Tandem stance, 60 s × 3 sets
	Hundred, 1 set	Single leg balance, 60 s × 3 sets
	Roll over, 5 reps	Single leg swings (sagittal plane), 30 reps × 3 sets
	Double leg stretch, 5 reps	Single leg swings (frontal plane), 30 reps × 3 sets
	Double leg circle, 5 reps	Standing single leg circle, 30 reps × 3 sets
	Shoulder bridge, 5 reps in each leg	Single leg squat, 30 reps × 3 sets
	Swimming, 50 reps	Single leg deadlift, 30 reps × 3 sets
6 weeks	Abdominal prep, 10 reps	Balance equipment: BOSU (dome side)
	Rolling like a ball, 10 reps	Tandem stance, 60 s × 3 sets
	Hundred, 1 set	Single leg balance, 60 s × 3 sets
	Roll over, 10 reps	Single leg swings (sagittal plane), 30 reps × 3 sets
	Double leg stretch, 10 reps	Single leg swings (frontal plane), 30 reps × 3 sets
	Double leg circle, 10 reps	Standing single leg circle, 30 reps × 3 sets
	Shoulder bridge, 10 reps in each leg	Single leg squat, 30 reps × 3 sets
	Swimming, 100 reps	Single leg deadlift, 30 reps × 3 sets

**Table 2:** General characteristics of participants.

Variables	PT (*n* = 26)	BT (*n* = 25)	*p*-Value
Age, years	21.8 ± 1.6	22.5 ± 1.5	0.407
Height, cm	176.1 ± 4.5	176.9 ± 4.6	0.484
Weight, kg	69.1 ± 3.9	68.7 ± 3.5	0.824
BMI, kg/m^2^	22.3 ± 1.3	21.9 ± 1.5	0.537
LAS or giving way in the last week, *n*	1.3 ± 0.3	1.7 ± 0.5	0.256
LSA or giving way in the last month, *n*	3.9 ± 1.2	4.3 ± 1.0	0.435

*p* < 0.05; Abbreviations: PT, Pilates training; BT, balance training; BMI, body mass index; LAS, lateral ankle sprain.

**Table 3:** Isokinetic ankle strength test (30°/s).

		Isokinetic Strength Values				LSI (%)		
Variables	Group	Pre	Post	*p*-Values	Time × Group *p*-Values	Pre	Post	*p*-Values
Inversion, Nm	PT	42.2 ± 11.4	45.5 ± 18.4	0.513	0.247	90.5	92.2	0.419
	BT	45.9 ± 11.9	46.4 ± 16.3	0.312		89.3	90.7	0.598
Eversion, Nm	PT	22.3 ± 8.0	42.3 ± 14.5	0.029	0.339	57.9	88.2	<0.001
	BT	20.3 ± 9.3	49.2 ± 12.1	0.012		54.7	85.7	<0.001
Plantarflexion, Nm	PT	106.2 ± 21.0	112.1 ± 21.8	0.210	0.470	90.8	93.7	0.468
	BT	98.8 ± 18.8	109.9 ± 23.6	0.249		91.6	92.6	0.513
Dorsiflexion, Nm	PT	21.2 ± 9.7	33.6 ± 5.9	0.014	0.321	63.9	87.1	<0.001
	BT	20.8 ± 9.1	35.2 ± 6.0	0.019		66.1	86.4	<0.001

*p* < 0.05; Abbreviations: PT, Pilates training; BT, balance training; LSI, limb symmetry index; Nm, Newton meter.

**Table 4:** Results of the hop tests for Pilates and balance training groups.

		Single Hop Tests Values				LSI (%)		
Variables	Group	Pre	Post	*p*-Values	Time × Group *p*-Values	Pre	Post	*p*-Value
Single, cm	PT	119.3 ± 19.0	157.0 ± 14.8	<0.001	0.415	78.7	90.2	0.005
	BT	123.3 ± 18.2	148.3 ± 16.3	<0.001		82.5	87.6	0.009
Triple, cm	PT	418.7 ± 43.0	461.9 ± 41.8	0.009	0.017	83.3	93.4	<0.001
	BT	409.1 ± 41.5	438.0 ± 42.7	0.041		79.6	87.1	0.004
Crossover, cm	PT	390.1 ± 37.1	433.1 ± 39.1	0.007	0.233	75.4	86.3	0.007
	BT	388.1 ± 39.4	442.0 ± 38.3	0.015		80.9	88.9	0.008
6-m hop, s	PT	2.77 ± 0.15	2.12 ± 0.14	<0.001	0.462	72.3	91.2	<0.001
	BT	2.60 ± 0.13	2.09 ± 0.13	<0.001		74.2	92.7	<0.001

*p* < 0.05; Abbreviations: PT, Pilates training; BT, balance training; LSI, limb symmetry index; m, meter; s, seconds.

**Table 5:** Results of the Y-balance test for Pilates and balance training groups.

		Dynamic Balance Values				LSI (%)		
Variables	Group	Pre	Post	*p*-Values	Time × Group *p*-Values	Pre	Post	*p*-Values
Anterior, cm	PT	51.7 ± 14.3	68.2 ± 16.0	0.046	0.246	76.8	89.0	<0.001
	BT	52.0 ± 15.9	65.4 ± 17.8	0.037		78.2	90.2	<0.001
Posteromedial, cm	PT	61.5 ± 21.1	73.3 ± 19.0	0.014	0.015	79.9	86.6	0.005
	BT	64.1 ± 19.4	82.2 ± 20.2	0.015		82.1	92.4	<0.001
Posterolateral, cm	PT	63.3 ± 27.3	71.8 ± 20.8	0.041	0.008	81.3	87.1	0.006
	BT	62.4 ± 28.2	81.2 ± 23.1	0.027		80.5	91.4	<0.001

*p* < 0.05; Abbreviations: PT, Pilates training; BT, balance training; LSI, limb symmetry index.

**Table 6:** Results of the Foot and Ankle Outcome Score for Pilates and balance training groups.

Variables	Group	Pre	Post	*p*-Values	Time × Group *p*-Values
Pain	PT	74.0 ± 18.4	88.4 ± 8.5	0.005	0.417
	BT	77.6 ± 24.1	89.8 ± 9.7	0.004	
Symptoms	PT	72.1 ± 19.6	92.7 ± 6.6	<0.001	0.356
	BT	70.1 ± 18.4	95.3 ± 4.1	0.009	
ADL	PT	76.9 ± 15.1	89.6 ± 9.6	0.007	0.507
	BT	71.2 ± 16.3	89.3 ± 8.4	0.004	
Sports and recreation	PT	65.7 ± 21.8	88.1 ± 7.0	<0.001	0.348
	BT	60.6 ± 17.5	90.7 ± 7.4	<0.001	
QoL	PT	69.5 ± 14.6	88.5 ± 8.1	0.008	0.271
	BT	71.3 ± 16.7	85.2 ± 9.9	0.004	

*p* < 0.05; Abbreviations: PT, Pilates training; BT, balance training; ADL, activities of daily living; QoL, quality of life.

## Data Availability

The data are not publicly available for privacy or ethical reasons.
